# A Pro-social Pill? The Potential of Pharmacological Treatments to Improve Social Outcomes After Pediatric Traumatic Brain Injury

**DOI:** 10.3389/fneur.2021.714253

**Published:** 2021-08-19

**Authors:** Bridgette D. Semple, Ramesh Raghupathi

**Affiliations:** ^1^Department of Neuroscience, Monash University, Prahran, VIC, Australia; ^2^Department of Neurology, Alfred Health, Prahran, VIC, Australia; ^3^Department of Medicine, Royal Melbourne Hospital, The University of Melbourne, Parkville, VIC, Australia; ^4^Graduate Program in Neuroscience, Graduate School of Biomedical Sciences and Professional Studies, Drexel University College of Medicine, Philadelphia, PA, United States; ^5^Department of Neurobiology and Anatomy, Drexel University College of Medicine, Philadelphia, PA, United States

**Keywords:** immature, neurotrauma, brain development, oxytocin, neuroprotection, behavior, rodent

## Abstract

Traumatic brain injury (TBI) is a leading cause of injury-induced disability in young children worldwide, and social behavior impairments in this population are a significant challenge for affected patients and their families. The protracted trajectory of secondary injury processes triggered by a TBI during early life—alongside ongoing developmental maturation—offers an extended time window when therapeutic interventions may yield functional benefits. This mini-review explores the scarce but promising pre-clinical literature to date demonstrating that social behavior impairments after early life brain injuries can be modified by drug therapies. Compounds that provide broad neuroprotection, such as those targeting neuroinflammation, oxidative stress, axonal injury and/or myelination, may prevent social behavior impairments by reducing secondary neuropathology. Alternatively, targeted treatments that promote affiliative behaviors, exemplified by the neuropeptide oxytocin, may reduce the impact of social dysfunction after pediatric TBI. Complementary literature from other early life neurodevelopmental conditions such as hypoxic ischemic encephalopathy also provides avenues for future research in neurotrauma. Knowledge gaps in this emerging field are highlighted throughout, toward the goal of accelerating translational research to support optimal social functioning after a TBI during early childhood.

## Introduction

Persistent social deficits are common after traumatic brain injury (TBI) during childhood, and their impact on quality of life is increasingly recognized (1). Social cognition, or the ability to perceive, interpret and act upon social information, underlies social interactions, communication and adjustment. All of these components of social functioning may be affected by brain injuries across a wide spectrum of severities in pediatric populations (2, 3). With TBI being a leading cause of injury-induced disability in young children worldwide, social behavior impairments in this population are a significant challenge for affected patients and their families. Alongside neurocognitive deficits, post-TBI social problems contribute to the financial burden associated with TBI rehabilitation care; with health and rehabilitation costs estimated to total around $1 million per injured person in the United States across their lifetime (4–6).

While a TBI sustained at any age has the potential to impair psychosocial function, the pediatric injured brain appears to be particularly susceptible to social behavior deficits. This vulnerability may be attributed to an immature state at the time of injury, such that injury disrupts not only the developing neural networks that underpin social cognition, but also the acquisition of new social skills (7, 8). Social deficits may persist and develop over time post-injury, and are often concomitant with cognitive problems, executive function and attention deficits (9). Longitudinal neuroimaging studies consistently show that alterations in brain structure and function can persist for an extended period of time after pediatric TBI, suggesting a link between progressive neuropathology and functional impairments over time (10). This protracted trajectory of secondary injury, alongside ongoing developmental maturation, offers a potential window of time during which external factors such as rehabilitation or drug treatments may yield functional benefit.

Rehabilitation for survivors of TBI is both multifaceted and interdisciplinary, and broadly aims to facilitate neurocognitive and functional recovery (11). Support for social cognition and social competence is typically embedded in this context, striving toward functional independence and reintegration into social networks, school and the workplace. The early initiation of rehabilitation therapies as well as an interdisciplinary model of care is important to maximize recovery for children with severe TBI (12, 13). However, few studies have examined the effectiveness of interventions on social impairments specifically. There is also considerable scope for complementary approaches to enhance the success of both social and cognitive rehabilitation, such as through pharmacological targeting. This may be *via* the administration of compounds that provide broad neuroprotection—for example, by targeting a range of secondary injury mechanisms that underpin progressive neuropathology and the development of social deficits. Alternatively, targeted treatment with drugs known to promote affiliative behaviors may be effective at reducing the impact of social dysfunction after TBI.

This mini-review describes the current state-of-the-field in the development of such therapies, with a focus on pre-clinical modeling in pediatric TBI. Drugs and targets with demonstrated potential in other early life neurodevelopmental disorders such as hypoxic-ischemic (HI) injury are also described where relevant. Knowledge gaps are highlighted throughout, and our goal is to drive toward accelerated translational research to support the optimal social functioning after pediatric TBI.

## Experimental Models Of Social Behavior Impairments After Tbi

Historically, the pre-clinical neurotrauma field has focused on assessments of sensorimotor and cognitive outcomes (14). Over the past decade, the negative impact of psychosocial impairments on quality of life has spurred an increase in pre-clinical studies incorporating measures of social behaviors. Several different paradigms to assess social investigation, social recognition and memory, and sociosexual interest are now established, as described in detail elsewhere (3, 15, 16). Social impairments typically manifest as a reduction in social investigation of a novel, unfamiliar conspecific either in an open field arena, home cage of the experimental animal, or the three-chamber social approach test, with the latter paradigm also allowing for the evaluation of social memory (reflecting social recognition) (17). These tests have largely been developed in models of disorders of neurodevelopment, such as autism spectrum disorders (ASD) of both genetic and acquired origins (18–21).

Semple and Noble-Haeusslein in 2012 first employed such methods to investigate social behavior changes in a model of severe TBI in mice at postnatal day (p) 21. Male mice were found to exhibit normal social behaviors at 2 weeks post-injury, approximately adolescence—but showed aberrant social interactions and social recognition memory by early adulthood (around 8 weeks post-injury) (22). Similarly, severe TBI in p14 rats led to deficits in social interaction and social novelty in adolescence (23). More recently, Runyan et al. reported that a moderate TBI in the p11 rat resulted in deficits in social recognition memory at adolescence and adulthood in both male and female rats (24). A similar trajectory is commonly seen in patients after childhood TBI, where deficits may emerge and evolve with developmental maturation (25, 26). These findings support the prevailing hypothesis that early life TBI interferes with an individuals' ability to acquire and/or consolidate age-appropriate milestones in social cognition and social skills (27, 28). Thus, both pediatric mouse and rat models demonstrate good face validity, or similar observations to what is observed in the human condition.

Several pre-clinical neurotrauma studies have subsequently incorporated measures of social behavior into their study designs, considering how social functioning may be altered after injuries sustained across a lifespan (29–31). The three-chamber social approach test, and/or the classical resident-intruder paradigm, are the most commonly used and appear to be the most robust for both mice and rats. A description of these tasks, and findings in both pediatric and adult rodent models of TBI, are reviewed in detail elsewhere (3). In addition to rodents, social deficits have also been reproduced after experimental TBI in flies (32) and zebrafish (33). Rodent TBI models have also been tested for predictive validity; meaning that factors which are known to influence social behavior in humans have also been demonstrated to affect social deficits in experimental models. For example, greater deficits are typically reported with increased injury severity or repeated insults (34–36), as well as with comorbidities such as acute colitis (37) or delayed hypoxemia in adult TBI animals (38). These findings are in alignment with clinical reports that both the extent of, and persistence of, social behavior impairments are dependent upon injury severity; although impaired social cognition may present even after mild injuries (1, 2, 39, 40). Indeed, mild TBI in adolescent rats has been reported to alter social play behaviors, in females in particular (41); while other models of mild TBI (predominantly in adult rodents) have reported either subtle changes or normal social behavior (34, 42).

With this expanding body of literature characterizing social behavior changes after pediatric TBI, the field is poised to now trial novel therapies for their potential to rescue or prevent such deficits. Although the field remains in its infancy, this mini-review will highlight the few studies conducted to date in this context. Where appropriate, we have extended the scope to other early life insults such as HI injuries, modeling encephalopathy of pre-maturity or perinatal stroke depending on the nature of the insult and timing (43, 44). Other childhood conditions in which social behavior changes are a characteristic feature (such as ASDs) also provide enticing insights into potential new avenues for therapies. Therapeutic agents examined to date fall roughly into three main categories: neuropeptides, hormones, or modulators of neuroinflammation.

## Hypothalamic Neuropeptides As Mediators Of Social Affiliation

Oxytocin and vasopressin are evolutionarily conserved neuropeptides with important roles in the control and regulation of social behaviors (45). In mice, mutations in the oxytocin or oxytocin receptor genes manifest in social recognition deficits (46–48); whereas in humans, genetic variations in the oxytocin receptor gene are associated with individual variability in social behaviors (49). In contrast to these findings, moderate TBI in the neonatal rat did not reduce expression of mRNA for oxytocin in the paraventricular nucleus of the hypothalamus despite the presence of deficits in social recognition behavior (24). However, potential changes in protein levels of oxytocin after pediatric TBI were not investigated.

Modulation of both the vasopressin and oxytocin signaling pathways has generated promising findings to date as a means to improve social deficits in human conditions in which aberrant social behaviors are a feature, such as ASD (50, 51). For example, postnatal systemic administration of arginine-vasopressin in the valproic acid rat model of ASD alleviates social preference deficits in the three-chamber test, alongside a reduction in stereotyped behaviors (21). More abundant literature pertains to exogenous oxytocin administration, which consistently promotes pro-social, affiliative behaviors in rodent models of ASD [e.g., (52, 53)]. Therapeutic use of both vasopressin and oxytocin in this context has progressed into clinical trials, with promising reports that intranasal treatment can reduce social deficits and enhance adaptive behaviors in both children and adults with ASD (50, 51, 54).

Targeting oxytocin or its receptor has also demonstrated broad neuroprotection in the context of acquired prenatal and perinatal brain insults, including models of pre-maturity, fetal asphyxia, and fetal growth restriction (55–57). However, to the best of our knowledge, no studies to date have incorporated measures of social behavior outcomes. As such, the potential for oxytocin modulation to ameliorate social impairments in this context remains unknown. Instead, a reduction in brain damage in these models has been attributed to the modulation of microglia by oxytocin signaling (56), effects on the hypothalamic-pituitary-adrenal axis (58), and the enhancement of inhibitory postsynaptic currents in hippocampal neurons (57).

In pediatric TBI, Runyan et al. have recently investigated the potential of oxytocin treatment to ameliorate social behavior deficits following moderate TBI in p11 rats (24). Intranasal administration of oxytocin reduced deficits in social recognition in a dose-dependent manner at 4–5 weeks after injury (equivalent to adolescence); brain-injured animals receiving 60 μg of oxytocin at 30–45 min prior to behavior testing exhibited social recognition behavior similar to sham-injured rats. Interestingly, the same dose of oxytocin had minimal effects in sham-injured animals, suggesting that brain injury may alter the sensitivity of the oxytocin receptor. The observed deficits in social recognition memory were accompanied by a decrease in the frequency of spontaneous inhibitory currents within the medial prefrontal cortex and oxytocin was able to reverse this decrease, providing insight into mechanisms underlying these deficits.

## Exogenous Hormones To Normalize Aberrant Social Behavior

A wide range of social behaviors including parental care, social interactions, play, aggression, and sexual behaviors, are influenced by gonadal hormones, including testosterone, estradiol and progesterone (59). Neuroendocrine dysfunction is a common long-term symptom following TBI, particularly in pediatric populations (60–62). Greco et al. first reported both acute and chronic deficits in testosterone after repeated mild TBI in adolescent male rats, which were associated with dysfunctional sociosexual behaviors (63, 64). However, much more research is needed to clarify the relationship between hormones and behavioral changes after early life injuries; which may subsequently pave the way for novel treatment targets (65).

Fundamental differences between sexes remain to be fully elucidated, with only one study in the p21 mouse reporting sex-specific phenotypes in social and sociosexual behaviors after severe TBI, as well as neuronal morphology in the prefrontal cortex and hippocampus, two brain regions with known roles in social functioning (66). As our appreciation grows for the many complex and varied ways that sex influences TBI outcomes (67), future pre-clinical studies should incorporate both males and females to more thoroughly delineate potential sex-based differences in social outcomes.

The potential for hormonal manipulation to modulate social dysfunction after brain injury can be gleaned from models of HI injury in the rodent. The p10 rat exhibits a reduction in same-sex social play behaviors in both male and female injured rats at 4–5 weeks post-injury (68). However, early post-injury administration of estradiol to increase circulating hormonal levels was found to restore normal play behaviors. This benefit is likely to be consequential to a broad range of mechanistic effects of estradiol in the injured brain, following reports in other studies that it can reduce histopathology in perinatal HI models by decreasing cell death, promoting cell genesis and enhancing neurotrophic and anti-inflammatory responses (69).

Finally, the steroid hormone progesterone has been extensively studied in models of adult TBI for its multiple mechanisms of purported neuroprotection (70, 71). Progesterone was found to reduce cognitive deficits and aberrant network hyperexcitability after TBI in the neonatal rat (72). In other models of TBI using juvenile rats or mice, progesterone is reported to ameliorate mitochondrial dysfunction, oxidative stress and spatial learning and locomotor deficits, the latter in a sex-specific manner, and with mixed effects on the extent of tissue loss (73–75). However, the potential effects of progesterone treatment on social behavior deficits after pediatric TBI have not yet been explored.

## Modulating Secondary Injury Processes: Neuroinflammation And Oxidative Stress

The neuroinflammatory response induced by a TBI has long been considered integral to functional and neuropathological outcomes (76, 77). While no therapies have yet been successfully translated into the clinic, a large number of pre-clinical studies have investigated whether modulation of neuroinflammation can promote improved neurobehavioral and functional outcomes after TBI [see review (78)].

In the pediatric injured brain, inflammation is similarly implicated in outcomes; however, several reports have demonstrated age-specific differences in the innate immune response in the immature injured brain (79–81). For example, the infiltration of neutrophils into the mouse brain after TBI at p21 is exacerbated compared to the adult, both in magnitude and time course (80). Neutrophil elastase (NE) is a destructive proteolytic enzyme released by infiltrating neutrophils upon activation, which promotes oxidative stress, cell death, extracellular matrix degradation, and perpetuation of neuroinflammation (82). Semple et al. (83) reasoned that NE may be a key determinant of secondary pathogenesis after TBI in the p21 mouse, and found that NE deficiency or inhibition attenuated vasogenic edema, neutrophil infiltration, oxidative stress and acute hippocampal cell death, which was associated with improvements in spatial memory retention and injury-induced hyperactivity. However, while deficits in sociability and social memory were observed in TBI mice, targeting NE was unable to rescue this phenotype (83).

Another promising therapeutic with widely touted neuroprotective and anti-inflammatory properties is erythropoietin (EPO). With a primary role in erythroid development and maturation during hematopoiesis, EPO is now well-known for additional effects on the central nervous system, ranging from stimulation of neurogenesis through to prevention of oxidative stress, inflammation and cell death (84, 85). Recent meta-analyses of EPO in clinical trials have reported that EPO may prevent mortality after TBI; however, whether EPO treatment can improve neurological and functional outcomes remains unclear (86–88). To our knowledge, no pre-clinical studies have considered the effect of EPO administration on social behavior outcomes after TBI. However, two studies of perinatal brain injury induced by uterine artery occlusion at embryonic day 18 in pregnant rats have tested early postnatal EPO treatment, either alone or in combination with melatonin. This insult caused hyperactivity and impaired social interactions in young rats (89). Postnatal EPO mitigated the social behavior abnormalities, alongside changes in neuroimaging suggestive of improved structural integrity and recovery of myelin (89); while EPO combined with melatonin normalized social interactions to sham levels (90).

Other potential therapeutic targets to alleviate social deficits have been revealed in models of perinatal or early postnatal brain insults. Adapting the well-established Rice-Vannucci model of HI injury in mice at either p5 or p10 (representing pre-term and term infants, respectively), Dupré et al. found that p5 injuries resulted in pronounced hyperactivity by adulthood, whereas injuries at p10 resulted in reduced social investigation (91). In contrast, mice deficient in tissue plasminogen activator (tPA) did not show such behavioral changes. The brains of tPA KO mice revealed a reduction in protease activity, IgG leakage and microglial activation, suggesting that dampening of inflammation may underlie the preservation of social function (91). Finally, components of the mammalian target of rapamycin (mTOR) pathway have been implicated in social behavior deficits after HI injury in neonatal rats (92). Activated by the phosphoinositide 3-kinase (PI3K) intracellular signaling pathway, mTOR and its downsteam targets are upregulated after unilateral carotid ligand and HI injury in p6 rats. The three-chamber test at p35 (adolescence) revealed HI-induced deficits in social novelty preference, alongside hyperactivity, with these abnormal behaviors being attenuated by post-injury treatment with the mTOR inhibitor everolimus (92).

It is noteworthy that several of these studies to date have detected social behavior deficits and hyperactivity concurrently in the same animals after injuries to the pre-term, term, pediatric or adult brain (83, 89, 91, 92). These phenotypes may correspond to the clinical setting where children after brain injuries often present with both attention-deficit hyperactivity disorder and social behavior problems (93, 94). Recent studies have suggested that aberrant social behaviors may be attributed, at least in part, to deficits in sustained attention and attentional control (95). As such the relationship between social functioning and attention in pediatric TBI warrants further investigation, with potential implications for novel therapeutic targeting of both comorbidities.

**Figure 1:**
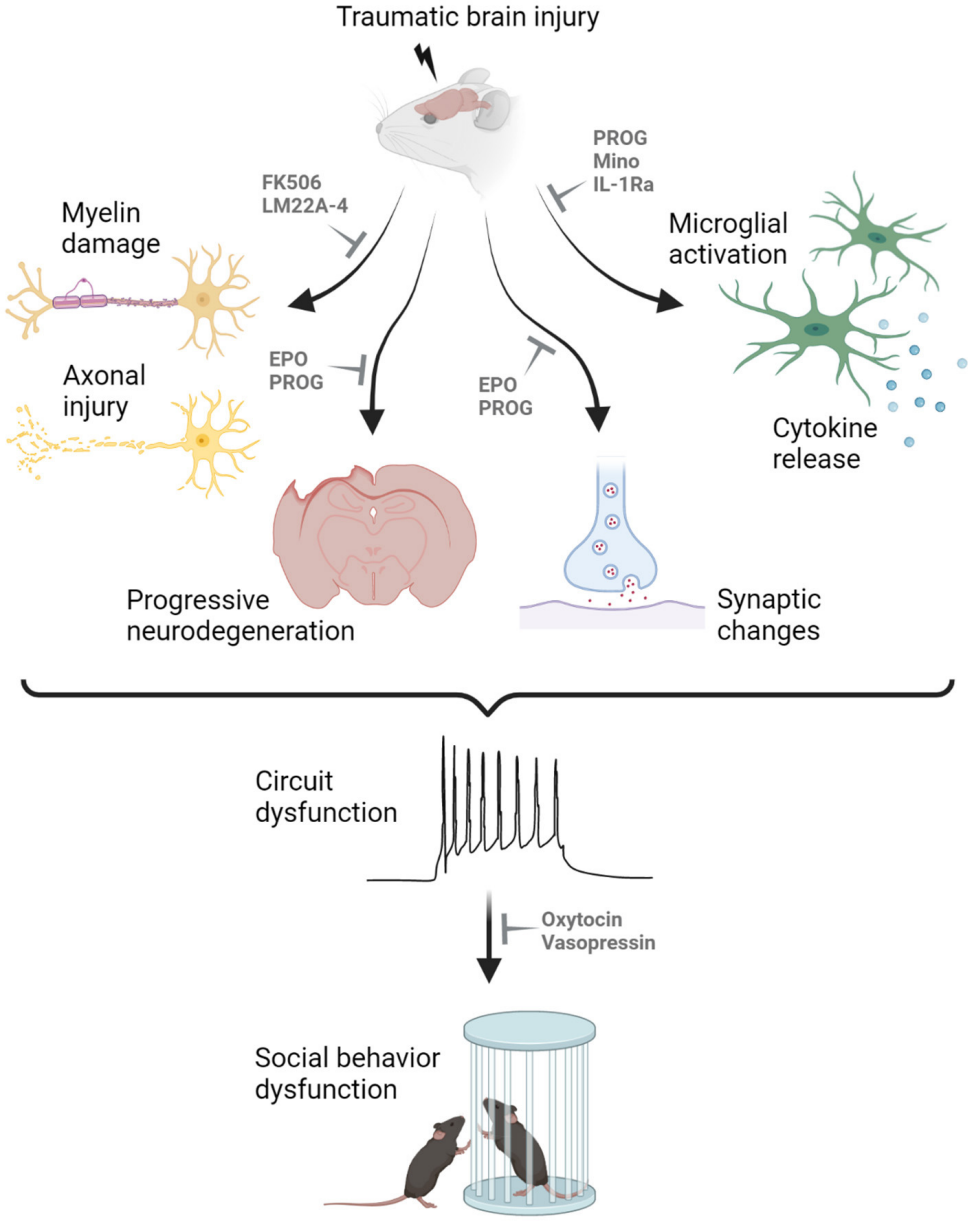
Proposed mechanisms underlying the manifestation of social behavior deficits after pediatric traumatic brain injury (TBI). Myelin damage, axonal injury, neurodegeneration, synaptic changes, microglial activation and cytokine release all contribute to the dysfunction of neuronal circuitry underlying social cognition, resulting in abnormal social interactions in rodent models of experimental TBI. Promising drug candidates to alleviated social behavior deficits are highlighted, targeting different secondary injury processes in the pediatric injured brain. For example, FK506 and LM22A-4 have been shown to reduce demyelination and axonal injury; progesterone (PROG) and erythropoietin (EPO) can prevent neurodegeneration and aberrant synaptic changes; and several compounds including PROG, minocycline (Mino) and interleukin-1 receptor antagonist (IL-1Ra) can minimize microglial activation and cytokine release. Such therapeutic targeting may reduce the extent of secondary injury after pediatric TBI to prevent the development of social behavior deficits. In contrast, treatments such as oxytocin and vasopressin may be administered once the abnormal circuitry is already present, to promote pro-social behaviors. Created with Biorender.com.

Broad neuroprotective agents with differing biological mechanisms may also influence social outcomes *via* a range of mechanisms, including neuroinflammation (Figure 1). In addition to those mentioned above, several other drugs have demonstrated promising neuroprotection in models of pediatric TBI, although the focus to date has been on sensorimotor and cognitive outcome measures. These include the calcineurin inhibitor FK-506 to reduce axonal degeneration (96); the TrkB agonist LM22A-4 to support myelination (97); minocycline to reduce microglia reactivity (98); and antagonism of the interleukin-1 receptor to reduce neuroinflammation and epileptogenesis (99). In this context, the goal is to prevent social behavior impairments from developing by reducing the extent of secondary brain damage after pediatric TBI. Progress in this field requires incorporation of social behavior assays in an increased proportion of pre-clinical TBI models going forward.

## Conclusion

In summary, pediatric TBI results in pronounced impairments in social interactions in rodent models, recapitulating a subset of aberrant social outcomes that are commonly observed after TBI in young children. Therapeutic targeting to improve social outcomes in experimental models are very limited to date. Findings regarding endogenous oxytocin treatment in the immature injured rat brain are promising (24), and several studies in models of early life HI provide avenues for future research in neurotrauma. Ultimately, the goals of such research should be two-fold: to both increase our understanding of the fundamental neurobiology underlying social impairments after pediatric TBI, and to identify novel therapeutic strategies that can ameliorate or prevent social behavior deficits. The continued characterization of social behavior impairments in pre-clinical models of pediatric TBI alongside neuropathological assessments, neuroimaging, and complementary neurobehavioral measures, is imperative for generating increased knowledge about the mechanisms that drive social deficits in this age group.

The combination of pharmacological targeting and rehabilitation strategies also deserves consideration. Although scarce, a few pre-clinical studies have evaluated the potential benefit of rehabilitation-based strategies in the aftermath of an early-life TBI; although social outcomes have not been evaluated (100, 101). Further, Kline et al. have reported that the combination of environmental enrichment and selected pharmacotherapies may have benefit above and beyond that of single therapies alone (102, 103). Thus, it is certainly feasible that complementary pharmacological and rehabilitation-based interventions may yield synergist benefits.

Altogether, the prospect of treating social behavior impairments with novel therapeutics after pediatric TBI is an exciting one, and we forecast significant advances in the field in the coming decade. Even incremental or subtle improvements in social functioning after pediatric TBI have the potential to significantly improve quality of life for survivors, through increased participation in society, peer friendships, family life, school and work activities.

## Author Contributions

BS and RR conceptualized and designed the manuscript. BS wrote the first draft, then BS and RR edited and revised the manuscript. Both authors approved the final submitted version.

## Conflict of Interest

The authors declare that the research was conducted in the absence of any commercial or financial relationships that could be construed as a potential conflict of interest.

## Publisher's Note

All claims expressed in this article are solely those of the authors and do not necessarily represent those of their affiliated organizations, or those of the publisher, the editors and the reviewers. Any product that may be evaluated in this article, or claim that may be made by its manufacturer, is not guaranteed or endorsed by the publisher.
